# Anti-IL-5 therapy in patients with severe eosinophilic asthma – clinical efficacy and possible criteria for treatment response

**DOI:** 10.1186/s12890-018-0689-2

**Published:** 2018-07-18

**Authors:** Nora Drick, Benjamin Seeliger, Tobias Welte, Jan Fuge, Hendrik Suhling

**Affiliations:** 1 Department of Respiratory Medicine, Hannover Medical School, Carl-Neuberg-Str.1, 30625 Hannover, Germany; 2 Biomedical Research in End-Stage and Obstructive Lung Disease Hannover (BREATH), Member of the German Centre for Lung Research (DZL), Hannover, Germany

**Keywords:** Severe eosinophilic asthma, Mepolizumab, Treatment response, IL-5, Lung function

## Abstract

**Background:**

Interleukin-5 (IL-5) antibodies represent a promising therapeutic option for patients with severe eosinophilic asthma. To date, no official treatment response criteria exist. In this study, simple criteria for treatment response applicable to all asthma patients were used to evaluate clinical efficacy and predictors for treatment response in a real-life setting.

**Methods:**

Data from 42 patients with severe eosinophilic asthma treated with mepolizumab for at least six months were analysed. Simple criteria to assess treatment response in clinical practice were used: increase of FEV_1_ ≥ 12% or ≥ 200 ml, reduction of blood eosinophils (< 150/μl or < 80% from baseline) and improvement of subjective condition (patient-judged subjective improvement or worsening following therapy). Patients were considered treatment responders if two criteria were fulfilled.

**Results:**

Thirty-two out of 42 patients (76% [61–87%]) were classified as responders. Within the groups (responder vs non-responder), treatment with mepolizumab led to significant increase in FEV_1_ (+ 600 ml vs -100 ml, *p* = 0.003), oxygenation (+ 8 mmHg vs -3 mmHg, *p* = 0.001), quality of life (visual analogue scale; + 28% vs − 5%, *p* = 0.004) and Asthma Control Test (+ 8 vs + 1 points, *p* = 0.002). In the responder group a significant decrease in the exacerbation rate over 12 months (1.45 vs 0.45, p = 0.002) was observed. Baseline characteristics (sex, BMI, smoking history, allergies, baseline level of eosinophils) did not predict treatment response.

**Conclusion:**

Using improvement of lung function, decrease of eosinophils and improvement of subjective condition as response criteria, 76% of treated patients could be classified as treatment responders, demonstrating the efficacy of anti-IL-5 therapy in clinical practice.

## Background

Asthma is a common chronic disease and affects approximately 315 million people worldwide [1]. About 3–10% of all asthma patients suffer from severe asthma which is defined as asthma remaining uncontrolled despite treatment with high-dose inhaled glucocorticoids combined with other controllers (long-acting β2-agonist, long-acting antimuscarinic agent, leukotriene receptor antagonist or theophylline) and/or treatment with systemic corticosteroids for at least 6 months [2, 3]. Despite the rather small percentage, patients with severe asthma are responsible for up to 50% of the direct and indirect costs associated with bronchial asthma [4]. Due to distinct symptoms, frequent exacerbations and numerous medication side effects, severe asthma represents a substantial burden for affected patients [5].

The first IL-5 antibody mepolizumab has been approved for over two years and has since become an established therapy for patients with uncontrolled severe asthma caused mainly by type 2 inflammation. Type 2 inflammation is characterized by the presence of IL-4, IL-5 and IL-13, produced by helper T cells leading to production, recruitment and activation of eosinophil granulocytes [6].

In large placebo-controlled trials, treatment with mepolizumab was well tolerated, resulting in a significant reduction of exacerbations and intake of oral corticosteroids (OCS) [7–9]. Meanwhile, another IL-5 antibody (reslizumab), differing from mepolizumab by the route of administration and an IL-5 receptor antibody (benralizumab) are available [10, 11]. Besides reduction of exacerbation rates and OCS dosages, all anti-IL-5 treatments led to a small but partly significant improvement of lung function [10, 12, 13]. As IL-5 functions as a central cytokine for activation and recruitment of eosinophils, it is not surprising that the number of eosinophil granulocytes in peripheral blood has been shown to be a predictor of clinical efficacy in targeted anti-IL-5 treatment with a greater reduction of exacerbations in patients with an eosinophil blood count of ≥150/μl [14]. So far this is the only available biomarker for selection of patients who are most likely to benefit from anti-IL-5 treatment [15]. The percentage of severe asthma patients presenting with high numbers of eosinophils is unknown, but studies of mild to severe asthma suggest approximately 50% [16]. Besides the initial studies which led to approval of the drugs, experience in clinical practice and efficacy is scarce. Especially, distinct definitions of treatment response to anti-IL-5 therapy remain to be elaborated. The *National Institute for Health and Care Excellence* (NICE) has published recommendations, defining the reduction of the exacerbation rate by at least 50% or a clinically reduced dose of continuous OCS as adequate response [17]. These criteria are not applicable to all patients with severe asthma, as not all patients require continuous OCS treatment or suffer from frequent exacerbations. We propose treatment response criteria, which are easy to assess and applicable to all patients as a continuous OCS therapy as well as frequent exacerbations are not required. Based on our treatment response criteria, we report the clinical efficacy of anti-IL-5 treatment in real-life setting and analyse potential predictors for treatment response.

## Methods

In this single-centre, retrospective analysis, clinical efficacy of IL-5 antibody therapy with mepolizumab and potential predictors for treatment response in patients with severe eosinophilic asthma were examined. All patients were treated with high-dose inhaled glucocorticoids and a long-acting β2-agonist, partially with a second or third controller and partially with additional OCS therapy. Documentation of eosinophil counts of ≥300 cells/μl in peripheral blood within the past 12 months had to be present. All patients included received mepolizumab subcutaneously once every 4 weeks for at least 6 months. All patients under follow-up at our asthma outpatient clinic provided written informed consent and all retrospective analyses were performed with approval of the local institutional review board.

### Treatment response criteria

According to the following treatment response criteria, patients were divided into two groups: responder and non-responder. To be classified as responder, at least two out of the three criteria had to be fulfilled. According to the *Global Initiative for Asthma* (GINA) the long-term goal of asthma treatment is represented by the control of symptoms and reduction of disease burden. In comparison to patients with mild Asthma, patients with severe Asthma face additional burdens influencing quality of life such as medication side effects, comorbidities or severe exacerbations leading to hospitalization [18]. To include all different aspects influencing daily life of patients with severe asthma, we included the overall term of *improvement of subjective condition* as the primary criterion. During interview patients were asked by the physician whether their subjective condition under therapy had improved or worsened (yes / no question). For their answer, patients were asked to consider asthma-related symptoms, quality of life (QoL), number of exacerbations and improvement of physical fitness.

Improvement of lung function is one central aspect of bronchial asthma therapy and for anti-IL-5 therapies improvement of FEV1 could be shown [19]. Therefore, improvement of lung function presents the second treatment response criterion (increase of forced expiratory volume in one second - FEV1 ≥ 12% or ≥ 200 ml). Values were chosen by analogy to the cut-offs used by the *Global Lung Initiative* [20].

Higher levels of eosinophils correlate with degree of airflow obstruction and disease severity as demonstrated by *Hancox* et al. [21]. Further, in severe asthma the extent of reduction in sputum eosinophils correlated with better asthma control [22]. Given these observations, we selected reduction of eosinophils in peripheral blood as third criterion (decrease in peripheral eosinophil blood count < 150/μl or less than 80% from baseline, by analogy to the mepolizumab approval studies [23]).

### Follow-up and work-up

Routine follow-up in our outpatient clinic includes spirometry or body plethysmography standardized to ERS/ATS guidelines, blood gas analysis, and laboratory testing if indicated. Structured questionnaires, assessing for exacerbation rate, physical fitness (measured by flights of stairs), asthma control (Asthma Control Test - ACT), quality of life (EQ-5D-3 L and visual analogue scale [VAS]) and subjective condition are completed at each attendance. QoL was assessed using the EQ-5D-3 L visual analogue scale ranging from 0% (worst imaginable health state) to 100% (best imaginable health state) [24, 25]. The ACT consists of 5 questions assessing asthma control in the previous 4 weeks inquiring the following asthma-related symptoms and items: shortness of breath, use of rescue inhaler, awakening at night due to wheezing, cough, shortness of breath, chest tightness or pain, activity limitation and self-perception of asthma control. The score ranges on a scale from 1 (poorly controlled) to 5 (well controlled), with a maximum score of 25. The ACT cut-off for GINA-defined uncontrolled asthma is ≤19; the recommendation for patients with severe asthma is ≤16 [26]. Exacerbations were defined as worsening of asthma symptoms requiring OCS or an increase in the OCS dose.

### Assessment of treatment response

**Fig. 1:**
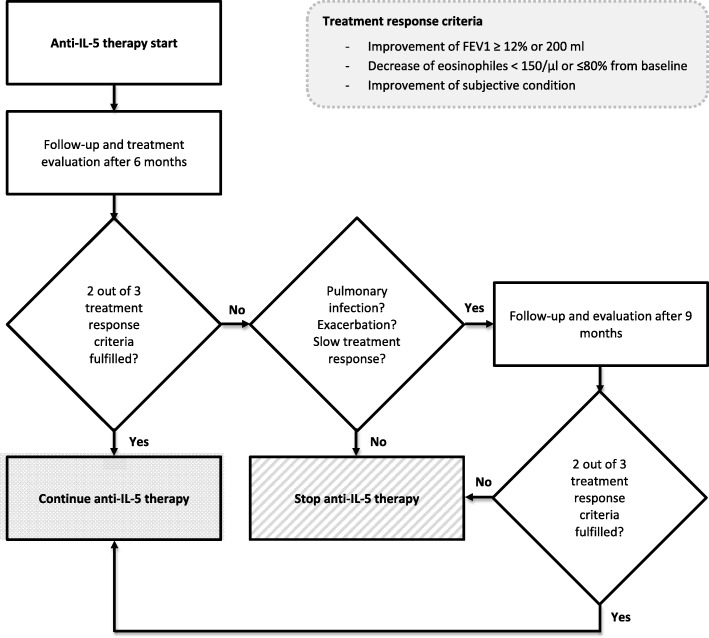
Follow-up after anti-IL-5 therapy initiation. FEV_1_ (forced expiratory volume in one second)

Data for analysis was assessed before treatment initiation (baseline) and at the latest follow-up appointment. The first follow-up appointment to assess treatment response was scheduled after 6 months. If responder criteria were not fulfilled, possible reasons for treatment non-responsiveness were evaluated. If the lack of response was attributable to an acute exacerbation or pulmonary infection or if the patient merely described a slow improvement but did not yet fulfil criteria, treatment was continued for another 3 months. If lack of response could not be explained by pulmonary infection or acute exacerbation and/or patients describe worsening of symptoms, IL-5 treatment was stopped. Follow-up evaluation is illustrated in Fig. 1.

### Statistical analysis

IBM SPSS Statistics 25.0 (IBM Corp, USA) and STATA 13.0 (State Corp LP, USA) statistical software were used for univariate analysis. Categorical variables are stated as numbers (n) and percentages (%). Continuous variables are shown as mean ± SD (if normally distributed) or median and interquartile ranges unless indicated otherwise. For group comparisons (responder vs non-responder), Fisher’s exact test, Chi-squared test, two-sided independent t-test or Mann-Whitney-U-test were used as appropriate. Logistic Regression models were created to determine effects on outcomes and in order to identify possible independent baseline characteristics having an effect on outcome, receiver operated characteristics (ROC) curves were drawn and the area under the curve (AUC) was calculated. All reported *p*-values are two-side. *P*-values < 0.05 were considered statistically significant. Dot-plots were created using Prism 7.04 (GraphPad, USA).

## Results

Data from 42 patients with severe eosinophilic asthma and treatment with mepolizumab were analysed. Patient characteristics with group differences are displayed in Table 1.

**Table 1:** Demographics at baseline

Characteristic	All (*n* = 42)		Anti-IL-5 therapy responder (*n* = 32)		Anti-IL-5 therapy non-responder (*n* = 10)		*p-value*
Age (years), median (IQR)	51	(45–59)	51	(44–60)	50	(44–58)	*0.456*
Female, *n* (%)	19	(45)	16	(50)	3	(30)	*0.267*
Allergies, *n* (%)	24	(57)	17	(53)	7	(70)	*0.347*
*Smoking history, n (%)*							
active smoker	1	(2)	1	(3)	0	(0)	
ex-smoker	20	(48)	14	(44)	6	(60)	
non-smoker	21	(50)	17	(53)	4	(40)	*0.432*
Pack years, median (IQR)	20	(10–33)	15	(7–35)	20	(10–20)	*0.708*
Body mass index, median (IQR)	28	(24–34)	28	(24–31)	31	(27–36)	*0.078*
*Lung function, median (IQR)*							
FEV_1_% of predicted	56	(41–71)	55	(46–67)	69	(39–80)	*0.635*
FEV_1_ (l)	1.8	(1.4–2.4)	1.8	(1.3–2.3)	1.8	(1.5–2.7)	*0.322*
FVCex % of predicted	82	(72–98)		78 (71–93)	94 (	82–101)	*0.108*
RV % of predicted	136	(119–174)	139	(122–174)	122	(107–162)	*0.419*
TLC % of predicted	100	(95–110)	101	(94–111)	98	(93–107)	*0.737*
*Laboratory, median (IQR)*							
Blood eosinophils (%)	7	(5–10)	7	(5–10)	6	(4–12)	*0.589*
Eosinophils absolute (cells/μl)	0.6	(0.4–0.8)	0.6	(0.4–0.8)	0.5	(0.4–1.0)	*0.228*
IgE (IE/ml)	128	(77–1222)	123	(66–1487)	298	(84–1362)	*0.966*
Continuous OCS therapy prior to IL-5 therapy, *n* (%)	23	(57)	17	(53)	6	(60)	*0.703*
Number of exacerbations per year prior to anti-IL-5 therapy, mean (±SD)	1.60	(±1.70)	1.78	(±1.77)	1.0	(±1.33)	*0.208*

*BMI* body mass index, *OCS* oral corticosteroids, *IgE* immunoglobulin E, *FEV*_*1*_ forced expiratory volume in one second, *FVC* forced vital capacity, *RV* residual volume. For comparisons, Fisher’s exact test, Chi-squared test, Mann–Whitney U test or two-sided paired t-test were used as appropriate

### Treatment response criteria

Thirty-two out of 42 patients (76%) were classified as treatment responders (95% confidence interval 61–87%). Median treatment time evaluated in the responder group was 12 months [IQR 8–15]. An improvement of subjective condition could be seen in all responders. A decrease of eosinophils (< 150/μl or less than 80% of baseline) was found in all patients except one (97%). Improvement of lung function was seen in 26/32 patients (81%). Ten patients did not fulfil response criteria and were classified as non-responders, accordingly. Anti-IL-5 therapy in these patients was discontinued after a median time of 9 months [IQR 6–12]. In the non-responder group, a decrease of eosinophils was observed in 8/10 patients (80%). There was no reported improvement of subjective condition, nor measured improvement in lung function in the non-responder-group. Response criteria and characteristics at follow-up visit are shown in Table 2.

**Table 2:** Therapy status at follow-up

Characteristic	All (*n* = 42)		Anti-IL-5 therapy responder (*n* = 32)		Anti-IL-5 therapy non-responder (*n* = 10)		*p-value*
Anti-IL-5 therapy in month, median (IQR)	12	(7–15)	12	(8–15)	9	(6–12)	*0.146*
*Anti-IL-5 therapy response criteria, n (%)*							
lung function (FEV_1_)	26	(62)	26	(81)	0	(0)	
eosinophils	39	(93)	31	(97)	8	(80)	
subjective condition	32	(76)	32	(100)	0	(0)	
*OCS therapy after anti-IL-5 therapy initiation*							
Continuous OCS therapy at baseline – mg/d, median (IQR)	5	(5–10)	5	(5–10)	5	(5–12.5)	*0.821*
Continuous OCS therapy at follow-up, mg/d, median (IQR)	5	(4–12.5)	4.5	(3.3–5)	10	(5–17.5)	*0.080*
OCS therapy discontinued at follow-up, *n* (%)	10	(24)	9	(28)	1	(10)	0.240

*OCS* oral corticosteroids, *FEV*_*1*_ forced expiratory volume in one second. For comparisons, Fisher’s exact test, Chi-squared test, Mann–Whitney U test or two-sided paired t-test were used as appropriate

### Changes in lung function, oxygenation, asthma control and quality of life

**Fig. 2:**
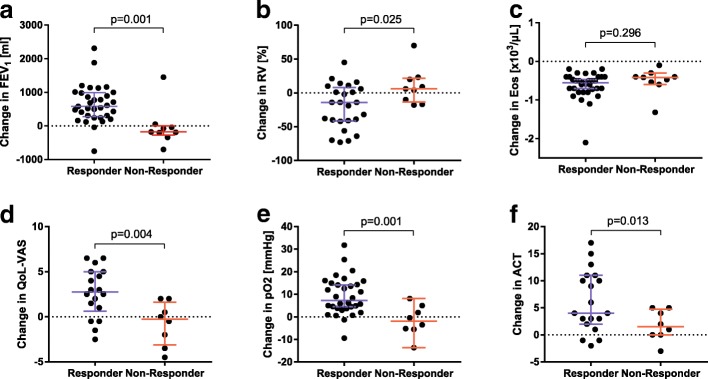
Comparison of the change (delta) from baseline to follow-up visit of lung function, blood eosinophils, capillary oxygenation, quality of life and asthma control test between both groups (responder vs non-responder). Percentages are stated as % of predicted. **a** FEV_1_, forced expiratory volume in one second; **b** *RV* residual volume; **c** *Eos* eosinophils; **d** *QoL* quality of life, *VAS* visual analogue scale; **e** *pO2* partial pressure of oxygen; **f** *ACT* asthma control test

In the responder group a significant improvement in lung function with increase of FEV_1_, increase of forced vital capacity (FVC) and decrease of residual volume (RV) at control visit could be shown compared to the non-responder group (Fig. 2). The FEV_1_ showed a median increase of 600 ml in the responder group whereas in the non-responder group a decrease of 100 ml could be measured (*p* = 0.003). Furthermore, oxygenation in capillary blood gas analysis was improved by 8 mmHg [IQR 4–15] in responder group and worsened by − 3 mmHg [IQR − 5-3] in the non-responder group (*p* = 0.001). Corresponding to the improvement of lung function and oxygenation patients in the responder group stated a significantly higher QoL according to VAS (improvement of 28% [IQR 6–50] vs. -5% [IQR -28-13], *p* = 0.004) as well as a significant higher score on the ACT compared to non-responder patients (improvement of 5 points [IQR 3–10] vs. 1 [IQR -2-5], *p* = 0.013). Within the responder-group, there was significant improvement in ACT, with a median of 12 points at baseline and 17 points at follow-up (*p* < 0.001). Non-responder patients improved by 1 point from 11 to 12 points.

### Exacerbation rates

In the responder group a significant difference in the exacerbation rate 12 months prior to anti-IL-5 therapy (1.45 ± 1.77) and post treatment initiation (0.45 ± 0.75) could be seen (*p* = 0.002). No prediction concerning the non-responder group could be made and no comparison between the responder and non-responder-group could be performed due to the small number of non-responder patients receiving anti-IL-5 therapy for 12 months.

### Oral corticosteroids

At baseline, 23/42 patients (57%) received continuous OCS, with similar dosages in both groups (5 mg [IQR 5–10] in the responder group, 5 mg [IQR 5–12,5] in the non-responder group). OCS dosages were reduced at follow-up in the responder group (4.5 mg [IQR 3.3–5]) and tended to increase in the non-responder group (10 mg [IQR 5–17.5], *p* = 0.080). Nine responder patients (28%) subsequently discontinued OCS versus one non-responder patient (10%, *p* = 0.240). Details are shown in Table 2.

### Prediction of treatment response

**Fig. 3:**
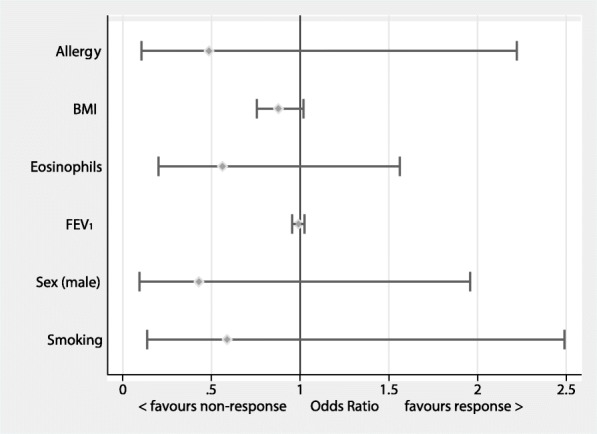
Analysis of potential predictors for treatment response. BMI, body mass index; FEV_1_, forced expiratory volume in one second

No significant differences could be shown between the groups concerning levels of eosinophils at treatment initiation with a median of 600 cells/μl in the responder group and 500 cells/μl in the non-responder group, respectively (*p* = 0.228). No significant differences between the groups could be shown concerning history of smoking (pack years), and existence of allergies or immunoglobulin E (IgE)-levels. There was no difference in the body mass index at treatment initiation (Table 1). Using univariate logistic regression, neither sex, body weight, IgE-level, level of eosinophils at baseline, allergy status nor lung function influenced allocation to treatment response groups (Fig. 3).

### Assessment of treatment response

At initial follow-up (median 6 months), 32/42 patients were grouped as responders and 10 patients did not fulfil response criteria; 4/10 patients without signs of acute exacerbation or pulmonary infection were classified as non-responders and therapy was discontinued. Of the remaining 6 patients who initially did not fulfil response criteria, therapy was discontinued due to lack of efficacy after 9 months (*n* = 4) and 12 months (*n* = 2).

### Side effects

In our cohort, no serious side effects leading to discontinuation of anti-IL-5 therapy occurred. The most common side effects were mild headache (7%), followed by injection-site reaction (5%), arthralgia (5%) and nausea (2%).

## Discussion

This study aimed to identify response criteria for treatment with mepolizumab and to evaluate clinical efficacy of anti-IL-5 therapy in clinical practice. Furthermore, potential predictors of treatment response were analysed.

Since the approval of mepolizumab as the first available IL-5 antibody, official treatment response criteria remain to be defined [6, 27]. Based on placebo-controlled phase III-studies [8, 9], recommendations published by NICE define the reduction of the exacerbation rate by at least 50% or a clinically significant reduced dose of continuous OCS as adequate response criteria [8, 9, 17]. In accordance with the literature, treatment with mepolizumab led to a significant reduction of asthma exacerbations in the responder group of our cohort. No prediction could be made concerning the definite exacerbation rate in non-responder patients as treatment in all but 2 patients was stopped before reaching the 12 months interval. As exacerbations mainly occur during winter, assessment of at least 12 months is mandatory to truly reflect the exacerbation rate [28]. Furthermore, exacerbations are common in patients with severe asthma, but are not an ubiquitous feature [29]. Therefore, while prevention of exacerbation represents a hallmark treatment goal, the exacerbation rate by itself hardly represents a valid criterion for treatment response in routine clinical practice. Similarly, only approximately 30% of patients with severe asthma are dependent on additional OCS treatment with many patients using OCS on demand rather than continuously [30]. In our cohort 55% of patients were receiving continuous OCS at baseline, rendering the reduction of OCS as an ineligible treatment response criterion in this study.

As the exacerbation rate as well as OCS treatment shows a close correlation to asthma-related QoL, we chose improvement of subjective condition (“subjective treatment response”) as one treatment response criterion [5, 31]. QoL and asthma control as traditional parameters were assessed separately with validated questionnaires, however, QoL is influenced by numerous aspects unrelated to asthma and the ACT has known limitations in severe asthma. *Korn* et al. recommended reducing the ACT cut-off for uncontrolled asthma in severe asthma to 16 [26]. Despite reduction of asthma-related symptoms following treatment, an ACT score remaining below 16 points indicates poorly controlled asthma where often there is no further option for therapy escalation. When asked about the effects of mepolizumab treatment, 32/42 patients (76%) in our cohort stated improvement of subjective condition. All 32 patients fulfilled at least two response criteria and were therefore grouped as responders, while in the non-responder group, no patient reported subjective improvement.

Given the type-2 immunologic pathway underlying eosinophilic asthma and the IL-5 antagonizing effects of mepolizumab, reduction of eosinophils as proof of interference with one main inflammatory mechanism was chosen as a second criterion for treatment response [32]. As IL-5 antibodies are approved for patients with blood eosinophils of ≥150 cells/μL at screening or ≥ 300 cells/μL within 12 months prior to treatment by the FDA (*Food and Drug Administration*) and EMA (*European Medicines Agency*), the blood eosinophil count had to be < 150 cells/μl or less than 80% from baseline to count as treatment responder in this category. A reduction to < 150 eosinophils or less than 80% from baseline could be seen in all responders except one. Interestingly, all non-responder patients except 2 dropped with eosinophil blood counts below 150/μl but did not show subjective improvement or improvement in lung function. This highlights that disease activity in refractory cases of eosinophilic asthma cannot be solely accounted for by eosinophilic inflammation. Some patients with severe asthma present with combined neutrophilic and eosinophilic inflammation of the airways detectable in sputum [33]. These patients with a phenotype of a mixed inflammatory response appear to have the greatest disease burden and airway limitation, necessitating further research for treatment strategies for neutrophilic or mixed inflammation in asthma [34].

*Price* et al. could show that patients with higher blood eosinophils are more likely to benefit from anti-IL-5 therapy but whether the statement “the higher the better” is true is not universally agreed [35]. Post-hoc-analysis of the phase III *calima* and *sirocco* studies showed that improvement of lung function in patients with benralizumab treatment was proportional to the extent of blood eosinophilia [36]. In the real-life scenario presented herein, this could not be reproduced by allocation to response groups as reduction of eosinophil count was present in both groups without significantly differing magnitude. As the blood eosinophil count represents the only available biomarker for initiation of anti-IL-5 therapy, little conclusion can be drawn from eosinophils regarding clinical response. Therefore, identification and validation of possible new biomarkers are highly desirable.

All patients with severe asthma at least intermittently show an impaired lung function with obstructive patterns. As improvement in lung function was frequently observed alongside subjective improvement and decline in eosinophil counts, we decided to include the improvement of lung function as a third response criterion using a slightly modified version of the approved bronchodilator criteria [37]. In our cohort 26 patients (79%) showed improvement of FEV_1_ of 12% or ≥ 200 ml. None of the non-responder patients showed improvement of lung function.

With these suggested response criteria 76% of our patients treated with mepolizumab could be classified as treatment responders. Treatment led to significant improvement in lung function, oxygenation, QoL and asthma control in responder vs. non-responder patients. Overall, anti-IL-5 therapy shows a favourable efficacy in patients with eosinophilic asthma in routine clinical practice.

Nevertheless, a quarter of patients with indication for anti-IL-5 therapy did not respond to treatment. In case of treatment failure, comorbidities as well as aggravating factors for severe eosinophilic asthma (such as allergic bronchopulmonary aspergillosis, bronchiectasis or eosinophilic granulomatosis with polyangiitis) should be ruled out. Demonstrated in family studies, there is increasing evidence that genetic abnormalities play a role in the pathogenesis of severe asthma [38]. Up to now, genetic testing is however not endorsed by international guidelines in these patients. Given the fixed mepolizumab dosage, under-dosing in obese patients should be considered, considering non-responders switching to intravenous anti-IL-5 agent allowing for individual dosing. Post-hoc analysis of the *Dose Ranging Efficacy And safety with Mepolizumab* (Dream)-study revealed similar reduction rates for exacerbations in obese and non-obese patients, but a recently published study demonstrated that in mepolizumab non-responders switch to intravenous, weight-adapted reslizumab can be beneficial [23, 39]. To account for overlap phenotypes with chronic obstructive pulmonary disease (COPD) and allergic bronchial asthma, we analysed smoking history and occurrence of allergies but no impact on treatment response was evident. Prior to anti-IL-5 therapy, 5 non-responder patients were eligible and received omalizumab therapy, but none of the patients responded to treatment. There was also no significant impact by body weight in our cohort.

Studies defining the appropriate follow-up schedule or the overall duration of anti-IL-5 therapy are missing. Mainly based on the approval studies, a treatment duration for initially 12 months is recommended due to the possibility of delayed treatment response [39]. In our cohort however, we did not observe any delayed treatment response in patients who failed to respond early after therapy initiation. Especially in regard to high treatment costs, regular assessment seems mandatory to detect treatment non-responders early [40]. When to delay or discontinue treatment with anti-IL-5 antibodies, is also still under debate. In patients with hypereosinophilic syndrome withdrawal of anti-IL-5 therapy led to a rebound of eosinophilia after 60–90 days [41]. In asthma patients a relapse of eosinophils to baseline could be seen after 6 months with a significant increase in exacerbations after 12 months [42]. Based on current available data, anti-IL-5 treatment should be regarded as long-term treatment.

### Limitations

There are important limitations to this analysis, mainly inherent by its retrospective design. This especially limits conclusion about exacerbation rates as exacerbations were only reported 12 months prior to treatment initiation. Value of the data and conclusions regarding the predictive power of the assessed factors are partly limited due to the small number of patients especially in the non-responder group. With the criterion of subjective treatment response, we used a non-validated assessment tool.

## Conclusion

Treatment with mepolizumab shows good efficacy and excellent safety in routine clinical practice. Using improvement of lung function, improvement of subjective condition and decrease of eosinophils in peripheral blood as treatment criteria, 3/4 of treated patients in our cohort can be classified as treatment responders. Anti-IL-5 therapy leads to significant increase in lung function, oxygenation, QoL and asthma control. Further research is needed to identify predictors for treatment response and to determine treatment duration.

## Abbreviations

ACT: Asthma control test
BMI: Body mass index
Eos: Eosinophils
FEV_1_: Forced expiratory volume in one second
FVC: Forced vital capacity
GINA: Global Initiative for Asthma
IgE: Immunoglobulin E
IL: Interleukin
NICE: National Institute for Health and Care Excellence
OCS: Oral corticosteroids
QoL: Quality of life
RV: Residual volume
VAS: Visual analogue scale
